# Optimized purification strategies for the elimination of non-specific products in the isolation of GAD65-specific monoclonal autoantibodies

**DOI:** 10.12688/f1000research.6467.2

**Published:** 2016-04-21

**Authors:** Wei Jiang, Henriette Macmillan, Anne-Marie Madec, Elizabeth D. Mellins

**Affiliations:** 1 Department of Pediatrics, Stanford University, Stanford, CA, 94305, USA; 2 Stanford Program in Immunology, Stanford University, Stanford, CA, 94305, USA; 3 Department of Medicine, University of California San Francisco, San Francisco, CA, 94143, USA; 4 INSERM U1060, Faculté de médecine Lyon-Sud, Oullins Cedex, France

**Keywords:** GAD65-specific, monoclonal autoantibody, affinity purification, autoantibody production

## Abstract

Autoantibodies against antigens expressed by insulin-producing β cells are circulating in both healthy individuals and patients at risk of developing Type 1 diabetes. Recent studies suggest that another set of antibodies (anti-idiotypic antibodies) exists in this antibody/antigen interacting network to regulate auto-reactive responses. Anti-idiotypic antibodies may block the antigen-binding site of autoantibodies or inhibit autoantibody expression and secretion. The equilibrium between autoantibodies and anti-idiotypic antibodies plays a critical role in mediating or preventing autoimmunity. In order to investigate the molecular mechanisms underlying such a network in autoimmunity and potentially develop neutralizing reagents to prevent or treat Type 1 diabetes, we need to produce autoantibodies and autoantigens with high quality and purity. Herein, using GAD65/anti-GAD65 autoantibodies as a model system, we aimed to establish reliable approaches for the preparation of highly pure autoantibodies suitable for downstream investigation.

## Introduction

Type 1 diabetes (T1D) is an autoimmune disorder characterized by the immune-mediated destruction of the insulin-producing β cells in the pancreas. Human islet cells express the 65-kDa isoform of glutamic acid decarboxylase (GAD65), which is one of the most common autoantigens associated with the development of T1D. Anti-GAD65 autoantibodies (GAD65Abs) are detectable several years before diabetes and present in over 70% of patients at the time of diagnosis ^1^. It has been suggested that healthy individuals also generate GAD65Abs, which are sufficiently neutralized by anti-idiotypic antibodies (anti-Id Abs), resulting in protection from GAD65-specific islet destruction ^2, 3^. Probably because the antigen-binding region of GAD65Abs is blocked by anti-Id Abs, circulating GAD65Abs in sera of healthy individuals are not detectable using GAD65-specific methods. The decline of anti-Id Abs in patients developing T1D, on the contrary, unmasks GAD65Abs, which then serve as critical serum markers in prediction and diagnostics of diabetes ^4^. Studies of the interaction between GAD65 and recombinant GAD65Abs have suggested immunodominant epitopes on GAD65 ^5– 9^. However, how the recognition of these epitopes by GAD65Abs drives islet destruction, and how anti-Id Abs block GAD65Ab-mediated auto-reactivity are largely unknown. In order to generate anti-Id Abs aimed at understanding of pathophysiologic mechanism(s), and more importantly, preventing GAD65 autoreactivity, it is necessary to isolate and utilize native GAD65Abs rather than synthesizing recombinant proteins. However, no published data have ever reported on the quality of purified GAD65Abs for such aims, even though two of these human Abs (b96.11 and b78) ^10– 13^ are commercialized.

Certain limitations stem from technical issues in the purification and characterization of native GAD65Abs originated from T1D patients. The most efficient way to produce monoclonal autoantibodies *in vitro* is to generate monoclonal B cell lines, culture them in batches, and purify the Abs from the culture supernatant. Although many established methods have been standardized for Ab purification ^14^, the polymorphic nature of Abs and the diverse culture conditions of Ab-secreting cell lines may impede the achievement of native autoantibody products with satisfactory quality and purity.

In this report, we evaluated multiple strategies for the purification of two human monoclonal GAD65Abs: DPA and DPD ^10^. Our goal was to isolate a pure population of Abs with minimal non-specific byproducts, in order to limit false positive results in downstream studies. We also determined GAD65-binding affinity of these two autoantibodies as the initial step of molecular characterization.

## Materials and methods

### Reagents

Detailed information on reagents used in this study is listed in Table 1.

**Table 1.** Details of reagents and materials.

Processes	Reagents and Materials	Manufacturers	Cat No.	Comments
Cell culture	IMDM	Life Technology	12440061	Complete IMDM includes 10% FBS, 2mM Glutamine, and 1% OPI
	Fetal Bovine Serum (FBS)	Atlanta Biologicals	S12450	
	L-Glutamine	Life Technology	25030	
	OPI Media Supplement	Sigma	O5003-1VL	
	AIM-V	Life Technology	12055	Serum-free
	BD cell Mab Medium	BD Biosciences	220509	Serum-free
RT-PCR	SuperScript III First-Strand Synthesis System	Life Technology	18080-051	
Ab purification	GammaBind Plus Sepharose	GE Healthcare	17-0886-01	
	nProtein A Sepharose	GE Healthcare	17-5280	
	Protein L Resin	GenScript	L00239	
	Vivapure Ion Exchange Spin Columns	Sartorius	VS-IX01QH24	Q Mini H Strong basic anion exchanger
		Sartorius	VS-IX01SH24	S Mini H Strong acidic cation exchanger
	Superdex 200 10/300 GL	GE healthcare	17-5175-01	
Gel electrophoresis	Mini-PROTEAN TGX Precast Gel	BioRad	456	4–15%, 4–20%, or 12% polyacrylamide gel
	2X Laemmli Sample Buffer	BioRad	161-0737	5% βME freshly added
Coomassie staining	SimplyBlue SafeStain	Life Technology	LC6065	
Western blotting	Immobilon-P Membrane	EMD Millipore	IPVH 00010	PVDF membrane
	Amersham ECL Western Blotting Detection Reagents	GE Healthcare	RPN2106	Reagents A and B
	Amersham Hyperfilm ECL	GE Healthcare	28-9068-39	
ELISA	NUNC 96 Well Flat-Bottom Immuno Plate, MaxiSorp,	Life Technology	442587	
	TMB Substrate Reagent Set	BD Biosciences	555214	Reagents A and B

### Cell lines

The monoclonal B cell lines secreting either DPA or DPD were immortalized by Epstein-Barr virus (EBV) transformation as described ^10^. These cell lines were maintained in complete Iscove’s modified Dulbecco’s medium (IMDM); or adapted to serum-free medium by diluting at a ratio of 1:2–1:3 every three days followed by a complete replacement after 10 days. Five million live cells were pelleted and reverse transcriptase polymerase chain reaction (RT-PCR) performed with the SuperScript III First-Strand Synthesis System (Life Technology) and antibody-specific primers ( Table 2).

**Table 2.** Oligonucleotides used in RT-PCR for cDNA verification.

Specific cDNA regions		Primer sequences
*IgG1 heavy chain*		
SN*	VH1	5’- CCCGAATTCATGGACTGGACCTGGAGG -3’
	VH2	5’- CCCGAATTCATGGACATACTTTGTACCAC -3’
	VH3	5’- CCCGAATTCATGGAGTTTGGGCTGAGC -3’
	VH4	5’- CCCGAATTCATGAAACACCTGTGGTTCTT -3’
	VH5	5’- CCCGAATTCATGGGGTCAACCGCCATCCT -3’
	VH6	5’- CCCGAATTCATGTCTGTCTCCTTCCTCAT -3’
ASN**	CTdomain***	5’- CTAGGCCCCCTGTCCGATCAT -3’
*κ light chain*		
SN	Vκ1	5’- CACAAGCCCAGCAACACCAAGGTGGAC -3’
	Vκ2	5’- GGGGGGAAGAGGAAGACTGACGGTCC 3’
	Vκ3	5’- GGGTGTACACCTGTGGTTCTCGGGGCTG 3’
	Vκ4	5’- GCAGGTGTAGGTCTGGGTGCC -3’
	Vκ5	5’- TGGCGGGAAGATGAAGACAG -3’
ASN	Cκ	5’- CTAAGACTCTCCCCTGTTGAA -3’
*λ light chain*		
SN	Vλ1	5’- CCCGAATTCATGGCCTGGGCTCCACTACT -3’
	Vλ2	5’- CCCGAATTCATGGCATGGATCCCTCTCTT -3’
	Vλ3	5’- CCCGAATTCATGGCCTGGGCTCTGCTGCTC -3’
	Vλ4	5’- ACCTATAAATATTCCGGATTATTCA -3’
	Vλ5	5’- TCTTGCCGGGTCCCAGG -3’
	Vλ6	5’- GGTCTCCAACAAAGCCCTCCC -3’
ASN	Cλ	5’- TTATGAACATTCTGTAGGGGCCACT -3’

* SN: sense primer. All SN primer sequences were described previously ^10^.

** ASN: antisense primer.

*** CTdomain: the oligonucleotide primes the 3’-end of the cytoplasmic tail of membrane Ig.

### Autoantibody purification

The supernatants of cell cultures containing Abs were filtered through a 0.22 μm membrane to remove cell debris. Abs were purified from the supernatant by affinity chromatography (as per manufacturer’s instructions ( Table 1)), followed by size exclusion chromatography (SEC) using a Superdex 200 gel filtration column (GE Healthcare). Fractions containing monomeric forms of each protein were pooled and analyzed by Coomassie stain or western blot.

### Coomassie staining and western blotting

Purified immunoglobulin G (IgG) products were reduced in sodium-dodecyl-sulphate (SDS) -containing Laemmli sample buffer with freshly added β-mercaptoethanol (βME) and denatured by boiling at 100°C for 10 min before separation by gel electrophoresis using Mini-PROTEAN TGX precast polyacrylamide gels (Bio-Rad). The gels were stained with SimplyBlue SafeStain (Life Technology) and destained with Milli-Q water for at least 1 h before imaging of IgG heavy and light chains. To differentiate the heavy and light chains of human IgG from non-specific contaminants co-purified from cell culture supernatant, proteins on the gel were transferred to Immobilon-P membrane (EMD Millipore) for human IgG detection. Goat F(ab’)2 anti-human Ig (2.5 mg/ml, Life Technology, Inc; used at 1:3000 dilution) followed by HRP-donkey anti-goat IgG (0.4 mg/ml, Santa Cruz Biotechnology, Inc; used at 1:10000 dilution) (see Table 3 for full Ab information) were used to detect human Ig.

**Table 3.** Abs generated or used in this report.

Antibodies	Manufacturers	Cat No.	RRID	Concentrations
DPA	IgG1 (VH4-DH-JH2)/λ(Vλ3-JL2), purified in this study			
DPD	IgG1 (VH4-DH-JH4)/κ(Vκ4-Jk4), purified in this study			
Goat F(ab’)2 anti- human Ig	Life Technology	H17000	RRID:AB_1500566	1:3000 for western blotting
HRP- Donkey anti- goat IgG	Santa Cruz Biotechnology, Inc.	sc-2020	RRID:AB_631728	1:10000 for western blotting
HRP- Goat F(ab’)2 anti-human Ig polyvalent	Life Technology	H17107	Discontinued	1:3000 for western blotting 1:20000 for ELISA
Mouse anti-GAD65 mAb, IgG1 isotype	Sigma	SAB4200232	RRID:AB_10762670	1:2000 for ELISA
HRP- goat anti-mouse IgG1 (γ1)	Life Technology	A10551	RRID:AB_10561701	1:2500 for ELISA

### Enzyme-linked immunosorbent assay (ELISA) for affinity measurement

Recombinant GAD65 (a gift from Peter van Endert, Institut National de la Santé et de la Recherche Médicale, France) in 20 mM 4-(2-hydroxyethyl)-1-piperazineethanesulfonic acid (HEPES)+1 mM pyridoxal phosphate (PLP)+50% Glycerol, pH 7.4, was stored in aliquots at -80°C; and the reactivity was verified by ELISA using commercially available mouse anti-GAD65 IgG1 and horseradish peroxidase (HRP) labeled goat anti-mouse IgG1 ( Table 3). The ELISA protocol for GAD65/autoantibody interaction has been described (see Table 4).

**Table 4.** ELISA protocol.

Steps	Reagents	Volumes	Conditions
Antigen Coating	GAD65 in 100 mM Carbonate-Bicarbonate Buffer pH 9.5	100 μl/well	4°C overnight
	PBS + 0.05% Tween 20	300 μl/well	Wash 3 times
Blocking	PBS + 2% BSA	250 μl/well	Room temperature (RT), 1 h
	PBS + 0.05% Tween 20	300 μl/well	Wash 3 times
Antibody Binding	Ab in PBS + 1% BSA	100 μl/well	37°C, 2 h
	PBS + 0.05% Tween 20	300 μl/well	Wash 5 times
Secondary Antibody Binding	HRP-Goat anti-hIg’s in PBS + 1% BSA	100 μl/well	RT, 1 h
	PBS + 0.05% Tween 20	300 μl/well	Wash 7 times
Detecting	TMB substrate A and B	100 μl/well	RT, 30 min
	1M Sulfuric acid	50 μl/well	Measure absorbance directly

## Results

**Figure 1.**
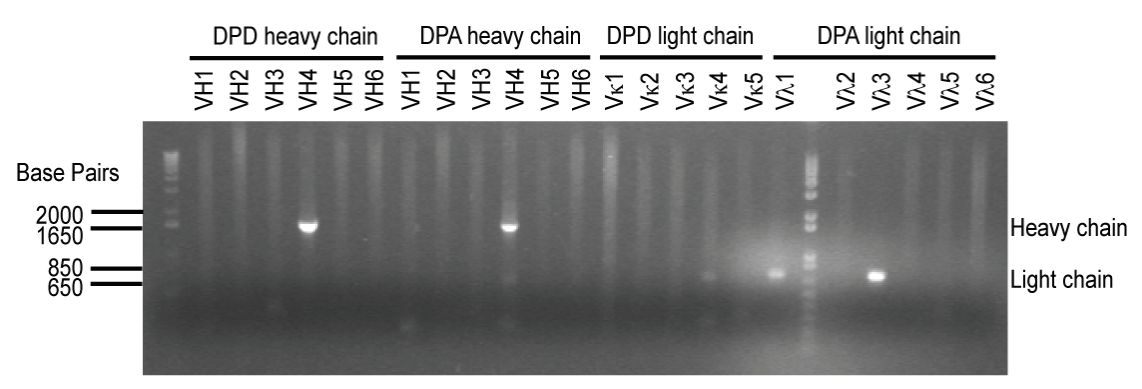
Ig cDNAs in monoclonal GAD65Ab-secreting cell lines. The RT-PCR amplified heavy chain cDNA using the indicated 5' primer and the 3' cytoplasmic-tail-specific primer, or the amplified light chain cDNA using the indicated 5' primer and the 3' constant-region-specific primer, are shown. Note that the PCR product amplified by Vλ1 from DPA-secreting cell line provided the same sequence as the one amplified by Vλ3, indicating that Vλ1 may result in non-specific primer annealing and PCR amplification.

Both GAD65Abs purified in this study belong to the human IgG1 (γ _1_) subclass; DPA uses a λ light chain and DPD uses a κ light chain ^10^. Prior to the purification of soluble DPA or DPD IgG from cell culture supernatant, we first validated Ig cDNA expression in each cell line using standard RT-PCR ( Figure 1). Note that we used an anti-sense oligonucleotide to prime the 3'-end of membrane IgG heavy chain cytoplasmic domain instead of one priming the 3'-end of the IgG heavy chain constant region used elsewhere, in order to generate the entire sequence of the heavy chain ( Supplementary file S1).

Although both anti-GAD65 Ab-secreting cell lines are derived from peripheral blood mononuclear cells (PBMCs) of a T1D patient ^10^, their culture conditions are significantly different. The DPA cell line expanded well in both serum-supplemented and serum-free medium, while the DPD cell line survived only in serum-supplemented medium. Fetal bovine serum (FBS) is widely used in tissue-culture medium to provide essential proteins, nutrients and other uncharacterized factors for optimum cell growth; however, the presence of bovine IgG (bIgG) in the serum (up to 50 mg/L) is the main source of contamination in human IgG (hIgG) purification. Bovine serum albumin (BSA) is also commonly used at a high concentration in culture medium (can be over 1 mg/ml), and binds non-specifically during the protein purification process.

**Figure 2.**
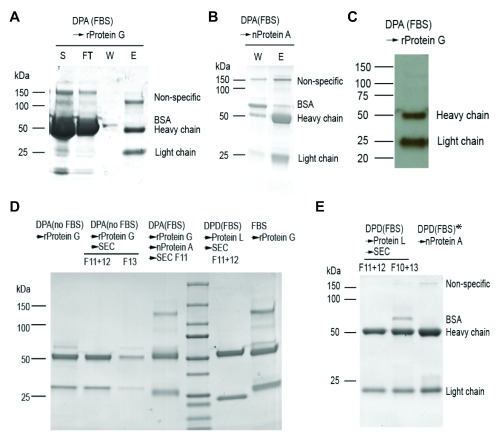
GAD65Abs purified using different methods. ( **A**, **B**) GAD65Ab-secreting cell lines were cultured with or without FBS, as indicated in parentheses, and the culture supernatant was applied to a pre-packed column containing one of the IgG-binding resins (right-pointing arrows) for affinity purification. Shown are Coomassie-stained gel images. S: supernatant; FT: flow through; W: wash; E: eluate. ( **C**) Western blotting analysis of eluted proteins from ( **A**) using anti-human Ig antibodies. ( **D**, **E**) Eluate from ( **A**) and ( **B**) was applied to a second column containing another IgG-binding resin or applied to a gel filtration column for size exclusion chromatography (SEC). Fractions (F) eluted from the gel filtration column were pooled before analysis by gel electrophoresis and Coomassie staining. Pure FBS was also applied to the gammabind resin-containing column for purification of bovine IgG. DPD (FBS)* indicates DPD culture supernatant pre-depleted with gammabind sepharose (Original gel images in Supplementary materials S2).

**Figure 3.**
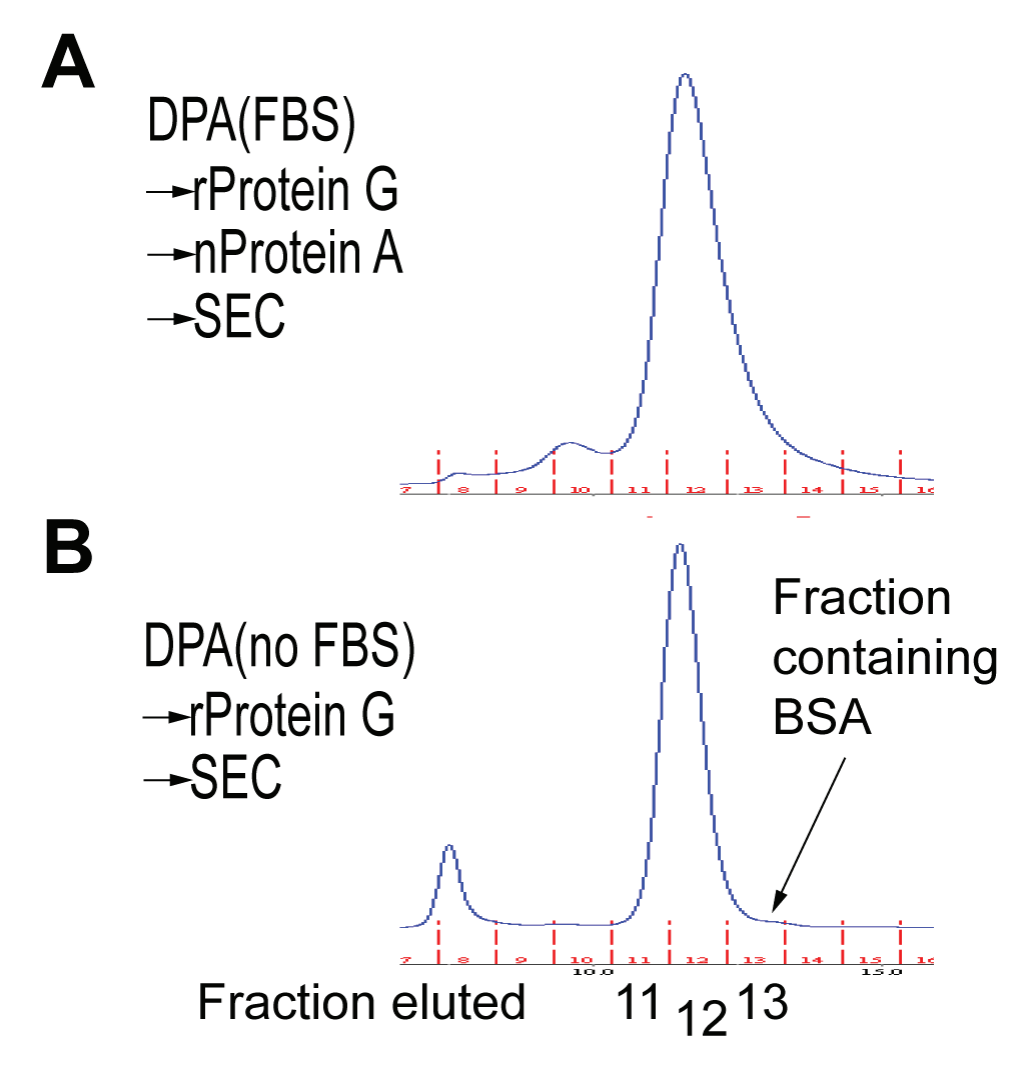
SEC profile of DPA with (A) or without (B) bovine IgG or BSA contaminants. ( **A**) DPA with bovine IgG eluted in more fractions (10–13 ml, 1ml per fraction), likely containing bIgG, unidentified bIgG-associated proteins, and BSA. ( **B**) Pure DPA without bIgG mainly eluted at two fractions (11 and 12 ml), which can be easily separated from BSA (~66.5 kDa, fraction 13) based on the difference in their sizes.

Affinity purification using antigens or IgG-binding proteins (e.g., Protein A, G and L) is very effective for Ab production, with antigen affinity purification being the most specific technique and providing the purest batches of antibody. However, GAD65Ab purification using recombinant GAD65 (rGAD65) for antigen-specific affinity purification is difficult because rGAD65 is unstable and requires pyridoxal phosphate (PLP) for stabilization. Considering the inevitable exposure of rGAD65 pre-coupled to resin to the extreme pH (<4 or >10) in elution and regeneration steps, this would not be a viable option. We therefore chose IgG-binding proteins in our attempt to affinity purify GAD65Abs without potential protein contaminants. Both Protein A and G recognize the Fc domain of IgG from human and bovine sera, while protein L binds to *κ* light chain. Gammabind sepharose beads (GE healthcare) use a recombinant form of Protein G (rProtein G), which significantly reduces the non-specific binding of BSA to the resin. Purification of IgG from the supernatant of DPA cell culture (grown in FBS-containing medium) on rProtein G resin resulted in purer IgG ( Figure 2A), than using native Protein A resin (nProtein A) ( Figure 2B). However, the purified IgG products from both rProtein G and nProtein A still contained a high molecular-weight (MW; MW>100 kDa) component besides the anticipated heavy chain (~50 kDa) and light chain (25 kDa) on coomassie-stained protein gels. Western blotting analysis suggested that this component did not belong to human Ig ( Figure 2C). The relative percentage of contamination with the high MW protein in IgG purified using nProtein A was significantly lower than when purified with rProtein G ( Figure 2A, Figure 2B). This component may reflect bIgG-associated contaminants, as bIgG has lower binding affinity for nProtein A than rProtein G. To test this, we gradually adapted DPA cells from FBS-containing medium to FBS-free medium and were able to affinity-purify hIgG from the culture supernatant without bIgG using rProtein G ( Figure 2D). We further separated DPA hIgG from any BSA contamination by SEC. The comparison between DPA purified using different methods and bIgG purified from pure FBS confirmed that the high MW contaminate is associated with bIgG ( Figure 2D and Figure 3). Importantly, we demonstrated that serum-free culture is key to isolating highly pure DPA hIgG.

**Figure 4.**
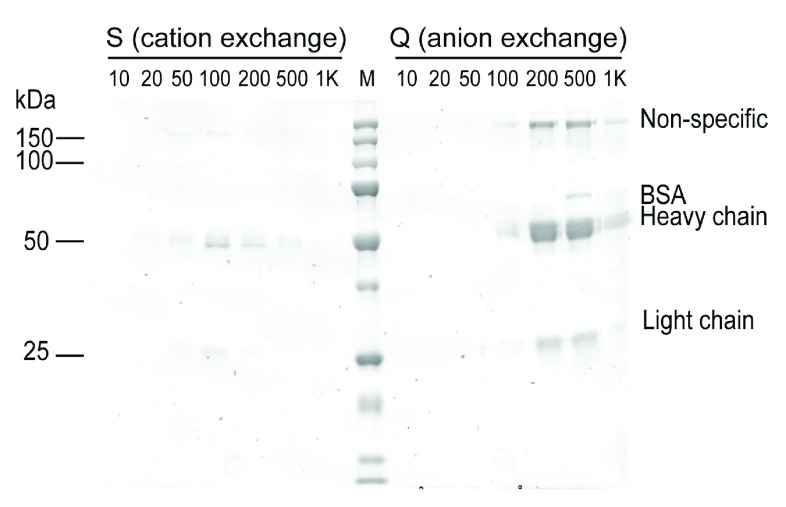
Ion exchange chromatography (IEC) of IgG purified from the DPA-secreting B cell line. Neither cation nor anion exchange separated hIgG from bIgG, as the non-specific bIgG associated band on the protein gel was present in all eluted fractions that contained IgG.

In contrast, DPD did not grow well in the serum-free medium we tested, and thus we opted to use Protein L as an alternative method to obtain more pure hIgG from this line. Protein L binds the light chain of IgG and DPD has a κ light chain. Notably, no previous evidence suggested that Protein L distinguishes κ chain of hIgG from bIgG; however, we found that Protein L affinity purification followed by SEC separation generated DPD hIgG with satisfactory purity and no detectable bIgG or bIgG-associated high MW proteins even though DPD cell culture contains 10% FBS ( Figure 2E). We also demonstrated that ion-exchange chromatography is not appropriate to separate hIgG from bIgG, as the high MW bIgG-associated protein(s) were present in all fractions eluted from anion or cation exchange columns ( Figure 4).

**Figure 5.**
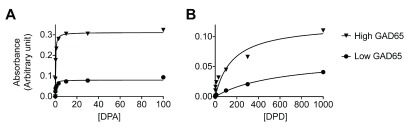
Binding of purified GAD65Abs to recombinant GAD65. 96-well plates were coated with two different concentrations of rGAD65 before incubation with different concentrations of ( **A**) DPA and ( **B**) DPD autoantibodies. The amount of GAD65Ab/rGAD65 complexes at equilibrium were measured by ELISA and plotted against the concentration of GAD65Abs. Data were fit to a single site binding equation for calculation of the dissociation constant.

We then determined the binding affinity of the purified DPA and DPD to rGAD65 by ELISA ( Figure 5). Given the instability of rGAD65, the measurement of its concentration was inaccurate. To overcome this problem, we coated the ELISA plate with two concentrations of rGAD65 (10–100 nM) differing by 3-fold and incubated immobilized rGAD65 with titrated amounts of purified DPA or DPD monoclonal Abs at 37°C for 2 h. The concentration of immobilized rGAD65 did not influence the calculation of the dissociation constant ( *K _D_*). We assumed that the duration of incubation was sufficient for the interaction between rGAD65 and GAD65Ab to reach equilibrium and fitted the data to a single site binding equation:

*y*= B _max_* *x*/( *K _D_*+ *x*)

to estimate *K _D_* ( Table 5). Purified DPA has over 100-fold higher rGAD65-binding affinity (the inverse of *K _D_*) than purified DPD.

**Table 5.** Fitting parameters and the dissociation constant.

mAb	Antigen	*B _max_* (Arbitrary unit)	*K _D_* (nM)	
			Fitted value	Estimated order of magnitude
DPA	Low GAD65	0.07982 ± 0.004650	0.3836 ± 0.1262	< 1
	High GAD65	0.3111 ± 0.005079	0.2624 ± 0.02543	
DPD	Low GAD65	0.6537 ± 0.007078	606.7 ± 136.8	> 100
	High GAD65	0.1155 ± 0.01786	136.2 ± 67.33	

## Conclusions

To the best of our knowledge, purification and characterization of native GAD65Ab, free from culture medium-derived contaminants such as bIgG and BSA, have not been reported previously, in spite of the availability of monoclonal cell lines secreting these Abs ^10, 11^. Our goal was to obtain a very pure preparation of GAD65-specific hIgG. Here, we demonstrate several strategies to overcome limitations associated with affinity purification that would be applicable to the purification of many other antibodies: (1) antigen-specific affinity purification is always superior, if the autoantigen itself can be easily produced and can tolerate exposure to pH extremes; (2) when dealing with an unstable autoantigen (most often), the attempt to adapt cells to serum-free medium is worthwhile to avoid bIgG contamination; (3) Protein L recognizes the light chains of Ig from different species, however, as we have shown here Protein L may preferentially bind human rather than bovine κ chain and provide an alternative approach to purification of autoreactive hIgG(κ).

It is of interest that there is over 100-fold difference in the rGAD65-binding affinity between DPA and DPD. Without understanding the mechanism, it is hard to predict the relationship between autoantigen binding affinity and the severity of disease. However, this finding reminds us that low affinity autoantibodies indeed exist, but are less likely to be detected in diagnostic tests, considering the binding of GAD65Abs by anti-Id Abs. Therefore, the detection threshold in diagnostic tests for measuring GAD65Abs or other autoantibodies in patient sera may need further optimization for a more thorough monitoring of low affinity autoantibodies and prediction of T1D.

## Supplementary Materials

### Supplementary file S1.

Sequences for human IgG DPA and DPD

### Supplementary materials S2.

Original gel images for Figure 2
