# Supplementary material for: Optimized purification strategies for the elimination of non-specific products in the isolation of GAD65-specific monoclonal autoantibodies
Source: F1000Res. 2016 Apr 21;4:135. Originally published 2015 May 29. [Version 2] doi: 10.12688/f1000research.6467.2 (PMC5680538; doi:10.12688/f1000research.6467.2)
Supplement: Supplementary file 1 [file f1000research-4-9048-s0000.tgz › f0cedf48-6a0d-48f2-b511-75395f741234.pdf]

DPA Heavy chain

```
      Q  V  Q  L  Q  E  S  G  P  G  L  V  K  P  S  E  T  L  S  L
1     caggtgcagctgcaggagtcgggcccaggactggtgaagccttcggagaccctgtccctc  60
      T  C  T  V  S  G  G  S  I  S  S  Y  Y  W  S  W  I  R  Q  P
61    acctgcactgtctctggtggctccatcagtagttactactggagctggatccggcagccc  120
      P  G  K  G  L  E  W  I  G  Y  I  Y  K  S  G  S  T  I  Y  N
121   ccagggaagggactggagtggattgggtatatctataagagtgggagcaccatctacaat  180
      P  S  L  K  S  R  V  T  I  S  V  D  T  S  K  N  Q  F  S  L
181   ccctccctcaagagtcgagtcaccatatcagtagacacgtccaagaaccagttctccctg  240
      K  L  T  S  V  T  A  A  D  T  A  V  Y  Y  C  A  R  E  P  P
241   aagctgacctctgtgaccgctgcggacacggccgtgtattactgtgcgagagagccaccc  300
      R  G  S  G  W  R  Y  W  Y  F  D  L  W  G  R  G  T  L  V  T
301   cggggcagtggctggcgatattggtacttcgatctctggggccgtggcaccctggtcact  360
      V  S  S  A  S  T  K  G  P  S  V  F  P  L  A  P  S  S  K  S
361   gtctcctcagcctccaccaagggcccatcggtcttccccctggcaccctcctccaagagc  420
      T  S  G  G  T  A  A  L  G  C  L  V  K  D  Y  F  P  E  P  V
421   acctctgggggcacagcggccctgggctgcctggtcaaggactacttccccgaaccggtg  480
      T  V  S  W  N  S  G  A  L  T  S  G  V  H  T  F  P  A  V  L
481   acggtgtcgtggaactcaggcgccctgaccagcggcgtgcacaccttcccggctgtccta  540
      Q  S  S  G  L  Y  S  L  S  S  V  V  T  V  P  S  S  S  L  G
541   cagtcctcaggactctactccctcagcagcgtggtgaccgtgccctccagcagcttgggc  600
      T  Q  T  Y  I  C  N  V  N  H  K  P  S  N  T  K  V  D  K  R
601   acccagacctacatctgcaacgtgaatcacaagcccagcaacaccaaggtggacaagaga  660
      V  E  P  K  S  C  D  K  T  H  T  C  P  P  C  P  A  P  E  L
661   gttgagcccaaatcttgtgacaaaactcacacatgcccaccgtgcccagcacctgaactc  720
      L  G  G  P  S  V  F  L  F  P  P  K  P  K  D  T  L  M  I  S
721   ctggggggaccgtcagtcttcctcttccccccaaaacccaaggacaccctcatgatctcc  780
      R  T  P  E  V  T  C  V  V  V  D  V  S  H  E  D  P  E  V  K
781   cggacccctgaggtcacatgcgtggtggtggacgtgagccacgaagaccctgaggtcaag  840
      F  N  W  Y  V  D  G  V  E  V  H  N  A  K  T  K  P  R  E  E
841   ttcaactggtacgtggacggcgtggaggtgcataatgccaagacaaagccgcgggaggag  900
      Q  Y  N  S  T  Y  R  V  V  S  V  L  T  V  L  H  Q  D  W  L
901   cagtacaacagcacgtaccgtgtggtcagcgtcctcaccgtcctgcaccaggactggctg  960
      N  G  K  E  Y  K  C  K  V  S  N  K  A  L  P  A  P  I  E  K
961   aatggcaaggagtacaagtgcaaggtctccaacaaagccctcccagcccccatcgagaaa 1020
      T  I  S  K  A  K  G  Q  P  R  E  P  Q  V  Y  T  L  P  P  S
1021  accatctccaaagccaaagggcagccccgagaaccacaggtgtacaccctgcccccatcc 1080
      R  E  E  M  T  K  N  Q  V  S  L  T  C  L  V  K  G  F  Y  P
1081  cgggaggagatgaccaagaaccaggtcagcctgacctgcctggtcaaaggcttctatccc 1140
      S  D  I  A  V  E  W  E  S  N  G  Q  P  E  N  N  Y  K  T  T
1141  agcgacatcgccgtggagtgggagagcaatgggcagccggagaacaactacaagaccacg 1200
      P  P  V  L  D  S  D  G  S  F  F  L  Y  S  K  L  T  V  D  K
1201  cctcccgtgctggactccgacggctccttcttcctctatagcaagctcaccgtggacaag 1260
      S  R  W  Q  Q  G  N  V  F  S  C  S  V  M  H  E  A  L  H  N
1261  agcaggtggcagcaggggaacgtcttctcatgctccgtgatgcatgaggctctgcacaac 1320
      H  Y  T  Q  K  S  L  S  L  S  P  E  L  Q  L  E  E  S  C  A
1321  cactacacgcagaagagcctctccctgtccccggagctgcaactggaggagagctgtgcg 1380
      E  A  Q  D  G  E  L  D  G  L  W  T  T  I  T  I  F  I  T  L
1381  gaggcgcaggacggggagctggacggctgtggacgaccatcaccatcttcatcacactc 1440
      F  L  L  S  V  C  Y  S  A  T  V  T  F  F  K  V  K  W  I  F
1441  ttcctgttaagcgtgtgctacagtgccaccgtcaccttcttcaaggtgaagtggatcttc 1500
      S  S  V  V  D  L  K  Q  T  I  I  P  D  Y  R  N  M  I  G  Q
1501  tcctcggtggtggacctgaagcagaccatcatccccgactacaggaacatgatcggacag 1560
      G  A  *
1561  ggggcctag  1569
```

DPA Light chain

```
     Q  S  A  L  T  Q  P  A  S  V  S  G  S  P  G  Q  S  I  T  I
1    cagtctgccctgactcagcctgcctccgtgtctgggtctcctggacagtcgatcaccatc  60
     S  C  T  G  T  S  S  D  V  G  G  Y  N  Y  V  S  W  Y  Q  Q
61   tcctgcactggaaccagcagtgacgttggtggttataactatgtctcctggtaccaacag  120
     H  P  G  K  A  P  K  L  M  I  Y  G  V  S  D  R  P  S  G  V
121  cacccaggcaaagcccccaaactcatgatttatggggtcagtgatcggccctcaggggtc  180
     P  D  R  F  S  G  S  K  S  G  N  T  A  S  L  T  I  S  G  L
181  cctgatcgcttctctggctccaagtctggcaacacggcctccctgaccatctctgggctc  240
     Q  A  E  D  E  G  D  Y  Y  C  S  S  Y  T  S  S  S  T  V  V
241  caggctgaggacgagggtgattattactgcagctcatatacaagcagcagcactgtggta  300
     F  G  G  G  T  K  L  T  V  L  G  Q  P  K  A  A  P  S  V  T
301  ttcggcggagggaccaagttgaccgtcctaggtcagcccaaggctgcccccctcggtcact  360
     L  F  P  P  S  S  E  E  L  Q  A  N  K  A  T  L  V  C  L  I
361  ctgttcccgccctcctctgaggagcttcaagccaacaaggccacactggtgtgtctcata  420
     S  D  F  Y  P  G  A  V  T  V  A  W  K  A  D  S  S  P  V  K
421  agtgacttctacccgggagccgtgacagtggcctggaaggcagatagcagccccgtcaag  480
     A  G  V  E  T  T  T  P  S  K  Q  S  N  N  K  Y  A  A  S  S
481  gcgggagtggagaccaccacaccctccaaacaaagcaacaacaagtacgcggccagcagc  540
     Y  L  S  L  T  P  E  Q  W  K  S  H  R  S  Y  S  C  Q  V  T
541  tatctgagcctgacgcctgagcagtggaagtcccacagaagctacagctgccaggtcacg  600
     H  E  G  S  T  V  E  K  T  V  A  P  T  E  C  S  *
601  catgaagggagcaccgtggagaagacagtggcccctacagaatgttcataa  651
```

DPD Heavy chain

```
      Q  V  Q  L  Q  E  S  G  P  G  L  V  K  P  S  E  T  L  S  L
1     caggtgcagctgcaggagtcgggcccaggactggtgaagccttcggagaccctgtcactc  60
      T  C  T  V  S  G  G  S  I  S  I  Y  Y  W  S  W  I  R  Q  P
61    acctgcactgtttctggtggctccatcagtatttactactggagctggatccgccagccc  120
      A  A  K  G  L  E  W  I  G  R  I  Y  T  N  G  T  T  N  Y  N
121   gccgcgaagggactggagtggattgggcgtatctataccaatgggaccaccaattacaac  180
      P  S  L  K  S  R  V  S  M  S  V  D  T  S  K  N  Q  F  S  L
181   ccctccctcaagagtcgagtctccatgtcagtagacacgtccaagaaccagttctccctg  240
      K  L  T  S  V  T  A  A  D  T  A  V  Y  Y  C  A  R  Q  G  G
241   aaactgacctctgtgaccgccgcggacacggccgtatattactgtgcgagacaagggggc  300
      L  V  G  A  S  G  R  R  D  Y  F  D  Y  W  G  Q  G  T  L  V
301   ctagtgggagccagcggacggcgtgactactttgactactggggccagggaaccctggtc  360
      T  V  S  S  A  S  T  K  G  P  S  V  F  P  L  A  P  S  S  K
361   accgtctcctcagcctccaccaagggcccatcggtcttccccctggcaccctcctccaag  420
      S  T  S  G  G  T  A  A  L  G  C  L  V  K  D  Y  F  P  E  P
421   agcacctctgggggcacagcggccctgggctgcctggtcaaggactacttccccgaaccg  480
      V  T  V  S  W  N  S  G  A  L  T  S  G  V  H  T  F  P  A  V
481   gtgacggtgtcgtggaactcaggcgccctgaccagcggcgtgcacaccttcccggctgtc  540
      L  Q  S  S  G  L  Y  S  L  S  S  V  V  T  V  P  S  S  S  L
541   ctacagtcctcaggactctactccctcagcagcgtggtgaccgtgccctccagcagcttg  600
      G  T  Q  T  Y  I  C  N  V  N  H  K  P  S  N  T  K  V  D  K
601   ggcacccagacctacatctgcaacgtgaatcacaagcccagcaacaccaaggtggacaag  660
      K  V  E  P  K  S  C  D  K  T  H  T  C  P  P  C  P  A  P  E
661   aaagttgagcccaaatcttgtgacaaaactcacacatgcccaccgtgcccagcacctgaa  720
      L  L  G  G  P  S  V  F  L  F  P  P  K  P  K  D  T  L  M  I
721   ctcctggggggaccgtcagtcttcctcttccccccaaaacccaaggacaccctcatgatc  780
      S  R  T  P  E  V  T  C  V  V  V  D  V  S  H  E  D  P  E  V
781   tcccggacccctgaggtcacatgcgtggtggtggacgtgagccacgaagaccctgaggtc  840
      K  F  N  W  Y  V  D  G  V  E  V  H  N  A  K  T  K  P  R  E
841   aagttcaactggtacgtggacggcgtggaggtgcataatgccaagacaaagccgcgggag  900
      E  Q  Y  N  S  T  Y  R  V  V  S  V  L  T  V  L  H  Q  D  W
901   gagcagtacaacagcacgtaccgtgtggtcagcgtcctcaccgtcctgcaccaggactgg  960
      L  N  G  K  E  Y  K  C  K  V  S  N  K  A  L  P  A  P  I  E
961   ctgaatggcaaggagtacaagtgcaaggtctccaacaaagccctcccagcccccatcgag 1020
      K  T  I  S  K  A  K  G  Q  P  R  E  P  Q  V  Y  T  L  P  P
1021  aaaaccatctccaaagccaaagggcagccccgagaaccacaggtgtacaccctgcccca 1080
      S  R  D  E  L  T  K  N  Q  V  S  L  T  C  L  V  K  G  F  Y
1081  tcccgggatgagctgaccaagaaccaggtcagcctgacctgcctggtcaaaggcttctat 1140
      P  S  D  I  A  V  E  W  E  S  N  G  Q  P  E  N  N  Y  K  T
1141  cccagcgacatcgccgtggagtgggagagcaatgggcagccggagaacaactacaagacc 1200
      T  P  P  V  L  D  S  D  G  S  F  F  L  Y  S  K  L  T  V  D
1201  acgcctcccgtgctggactccgacggctccttcttcctctacagcaagctcaccgtggac 1260
      K  S  R  W  Q  Q  G  N  V  F  S  C  S  V  M  H  E  G  L  H
1261  aagagcaggtggcagcaggggaacgtcttctcatgctccgtgatgcatgagggtctgcac 1320
      N  H  Y  T  Q  K  S  L  S  L  S  P  E  L  Q  L  E  E  S  C
1321  aaccactacacgcagaagagcctctccctgtctccggagctgcaactggaggagagctgt 1380
      A  E  A  Q  D  G  E  L  D  G  L  W  T  T  I  T  I  F  I  T
1381  gcggaggcgcaggacggggagctggacgggctgtggacgaccatcaccatcttcatcaca 1440
      L  F  L  L  S  V  C  Y  S  A  T  V  T  F  F  K  V  K  W  I
1441  ctcttcctgttaagcgtgtgctacagtgccaccgtcaccttcttcaaggtgaagtggatc 1500
      F  S  S  V  V  D  L  K  Q  T  I  I  P  D  Y  R  N  M  I  G
1501  ttctcctcggtggtggacctgaagcagaccatcatccccgactacaggaacatgatcgga 1560
      Q  G  A  *
1561  caggggcctag  1572
```

DPD Light chain

```
     D  I  V  M  T  Q  S  P  D  S  L  A  V  S  L  G  E  R  A  T
1    gacatcgtgatgacccagtctccagactccctggctgtgtctctgggcgagagggccacc  60
     I  N  C  K  S  S  Q  I  V  L  Y  S  S  N  N  K  N  Y  L  A
61   atcaactgcaagtccagccagattgttttatacagctccaacaacaagaactatttagct  120
     W  Y  Q  Q  K  P  G  Q  P  P  K  L  L  I  Y  W  A  S  T  R
121  tggtaccagcagaaaccaggacagcctcctaaactgctcatttactgggcatctacccgg  180
     K  S  G  V  P  D  R  F  S  G  S  G  S  G  T  D  F  T  L  T
181  aaatccggggtccctgaccgattcagtggcagcgggtctgggacagatttcactctcacc  240
     I  S  S  L  Q  A  E  D  V  A  V  Y  Y  C  Q  Q  Y  Y  T  T
241  atcagcagcctgcaggctgaagatgtggcagtttattactgtcagcaatactatactact  300
     P  L  T  F  G  G  G  T  K  V  E  I  K  R  T  V  A  A  P  S
301  ccgctcactttcggcggagggaccaaggtggagatcaaacgaactgtggctgcaccatct  360
     V  F  I  F  P  P  S  D  E  Q  L  K  S  G  T  A  S  V  V  C
361  gtcttcatcttcccgccatctgatgagcagttgaaatctggaactgcctctgttgtgtgc  420
     L  L  N  N  F  Y  P  R  E  A  K  V  Q  W  K  V  D  N  A  L
421  ctgctgaataacttctatcccagagaggccaaagtacagtggaaggtggataacgccctc  480
     Q  S  G  N  S  Q  E  S  V  T  E  Q  D  S  K  D  S  T  Y  S
481  caatcgggtaactcccaggagagtgtcacagagcaggacagcaaggacagcacctacagc  540
     L  S  S  T  L  T  L  S  K  A  D  Y  E  K  H  K  V  Y  A  C
541  ctcagcagcaccctgacgctgagcaaagcagactacgagaaacacaaagtctacgcctgc  600
     E  V  T  H  Q  G  L  S  S  P  V  T  K  S  F  N  R  G  E  S
601  gaagtcacccatcagggcctgagctcgcccgtcacaaagagcttcaacaggggagagtct  660
     *
661  tag  663
```
