# Supplementary material for: Optimized purification strategies for the elimination of non-specific products in the isolation of GAD65-specific monoclonal autoantibodies
Source: F1000Res. 2016 Apr 21;4:135. Originally published 2015 May 29. [Version 2] doi: 10.12688/f1000research.6467.2 (PMC5680538; doi:10.12688/f1000research.6467.2)
Supplement: Peer review history

# Peer review: Referee response for version 2

**DOI:** 10.5256/f1000research.9048.r16909

**Published:** 2016-10-25

**Authors:** Christiane Hampe

**Affiliations:** 1 Department of Medicine, Division of Metabolism, Endocrinology & Nutrition, University of Washington, Seattle, WA, USA

This is a very helpful paper, addressing in great detail problems involved in the purification of monoclonal antibodies for tissue culture supernatant and how to overcome these problems.

I have read this submission. I believe that I have an appropriate level of expertise to confirm that it is of an acceptable scientific standard.

# Peer review: Referee response for version 1

**DOI:** 10.5256/f1000research.6939.r9336

**Published:** 2016-03-10

**Authors:** Xiao He

**Affiliations:** 1 Department of Pathology, University of Utah, Salt Lake City, UT, USA

This is a good Ab validation study, where the authors testified multiple established Ab purification strategies in an autoimmune disease model. Non-specific proteins present in an autoantibody product could potentially affect its usage in many aspects. For example, the authors pointed out that pure autoantibodies can be more efficient in the study of both autoantigens and anti-idiotypic antibodies, although neither were further characterized. As this article mainly validated and discussed purification strategies, it will be more straightforward to emphasize only on Ab production, but not characterization, which was not the focus as I can tell.

I have read this submission. I believe that I have an appropriate level of expertise to confirm that it is of an acceptable scientific standard.

# Response

**Published:** 2016-04-22

**Authors:** Wei Jiang

**Affiliations:** Department of Pediatrics, Stanford University School of Medicine, USA

In light of Dr. He’s comment we have edited the title to “Optimized purification strategies for the elimination of non-specific products in the isolation of GAD65-specific monoclonal autoantibodies” in order to emphasize purification rather than characterization of the GAD65-specific antibodies. The abstract and introduction was also updated to reflect the focus on purification.

# Peer review: Referee response for version 1

**DOI:** 10.5256/f1000research.6939.r11219

**Published:** 2015-12-01

**Authors:** David Soll

**Affiliations:** 1 Department of Biology, University of Iowa, Iowa City, IA, USA

This article describes simple purification methods of highly pure antibodies from supernatant of human monoclonal B-cell cultures. However, all of the methods are already known. They are not new. One good thing that this article is showing is an example for the purification grades depending on the purification methods and culturing condition of the cells with and without FCS to avoid contamination with bovine antibodies. In my opinion, the abstract is not appropriated and overemphasized with points that are not directly related to this article.

Characterization of the antibodies is weak.

For the sentence on page 2 at the end of second paragraph “Utilization of impure GAD65Abs in the generation of anti-Id Abs and determination of their protective role in T1D pathogenesis may lead to unconvincing or inconclusive results.”, the authors have to explain with citations, why the purity of the antibody is important, and how the impure antibodies lead to unconvincing or inconclusive results.

I have read this submission. I believe that I have an appropriate level of expertise to confirm that it is of an acceptable scientific standard, however I have significant reservations, as outlined above.

# Response

**Published:** 2016-04-22

**Authors:** Wei Jiang

**Affiliations:** Department of Pediatrics, Stanford University School of Medicine, USA

Per Dr. Soll’s comment we have edited the title to “Optimized purification strategies for the elimination of non-specific products in the isolation of GAD65-specific monoclonal autoantibodies” to show that we have utilized known methods but we very specifically optimized the purification of the GAD65-specific antibodies.

The abstract and introduction was updated to focus on our goal of optimization and validation of approaches for the purification of highly pure autoantibodies. These approaches are not novel, but the specific application to purification of anti-GAD65 antibodies were never evaluated.

We agree that the characterization is weak and have updated the abstract and introduction to focus on purification. Highly purified autoantigen-specific antibodies could be suitable for the downstream study of mechanisms underlying the interaction between autoantigen and antibody or autoantibody and anti-idiotypic antibody which is beyond the scope of this current report.

We have removed the sentence on page 2 that read “Utilization of impure GAD65Abs in the generation of anti-Id Abs and determination of their protective role in T1D pathogenesis may lead to unconvincing or inconclusive results.” The purity of autoantibody might be important, for example, in the generation of anti-Id Abs; otherwise, there might be anti-byproduct Abs in the final mixture, which can be problematic. Since there are currently no publications reporting an issue caused by non-specific by-products in the production of anti-GAD65 autoantibodies we have removed the sentence.
